# Supplementary material for: Analyzing and predicting global happiness index via integrated multilayer clustering and machine learning models
Source: PLoS One. 2025 Apr 30;20(4):e0322287. doi: 10.1371/journal.pone.0322287 (PMC12043169; doi:10.1371/journal.pone.0322287)
Supplement: Attachment — Submitted filename: Response to Reviewers.docx [file pone.0322287.s003.docx]

**Response to Reviewers**

We sincerely appreciate the reviewers’ constructive feedback and have revised the manuscript accordingly. Below are our point-by-point responses:

**Academic Editor Comments**

1. **Manuscript Style Requirements**
   We have reformatted the manuscript to comply with PLOS ONE’s style guidelines, including adjusting section headings, citations, and file naming conventions. The structure now aligns with the journal’s template.
2. **Code Sharing Guidelines**
   Data and code upload.
3. **Copyrighted Figures**
   Figure 1 has been deleted.
4. **ORCID iD**
   The corresponding author’s ORCID (0009-0005-8959-868X) has been validated in Editorial Manager.
5. **Journal Template**
   The manuscript now follows the PLOS ONE template.
6. **Manuscript Structure**
   Sections have been restructured to include distinct “Related Work,” “Threats to Validity/Limitations,” and “Conclusion and Future Work” sections. The Methodology section now details each step systematically.
7. **Implications and Novelty**
   We respectfully believe that the manuscript already highlights the novelty of the study, which lies in integrating clustering analysis with machine learning models for enhanced prediction of the happiness index. We have explicitly discussed the implications of our findings for policy-making, particularly for countries with low happiness levels. Additionally, we have provided a detailed explanation of how our integrated methodology surpasses traditional approaches, offering a more nuanced understanding of global happiness.

**Reviewer #1 Comments**

1. **Vague Title & Abstract**
   *Comment:* The title and abstract use general terms and do not clearly state the unique contribution.
   *Response:* We appreciate the feedback. However, we believe the title and abstract clearly convey the key novelty of the study, which is the integration of clustering and machine learning techniques to enhance the prediction of the happiness index. The use of terms such as “hierarchical analysis” and “novel predictive framework” directly highlights the innovation and contributes to the overall clarity of the manuscript.
2. **Inconsistent Citation Style**
   *Comment:* Different citation styles used throughout the manuscript.
   *Response:* We have carefully reviewed and corrected the citation style to ensure consistency. The final version follows the required PLOS ONE citation style, and all references have been formatted accordingly.
3. **Non-Standard Sectioning**
   *Comment:* The manuscript structure does not follow a typical academic paper format, lacking a distinct “Literature Review” section.
   *Response:* While we understand the preference for a separate literature review, the introduction section in our manuscript serves this function by discussing key related works. We believe that integrating the literature review within the introduction enhances the flow of the narrative, but we have added clearer section headings to improve structure and readability.
4. **Inadequate Related Work**
   *Comment:* The “Related Work” section is too short and does not adequately relate to study goals.
   *Response:* We respectfully disagree with this assessment. We have thoroughly reviewed the relevant literature on clustering, machine learning for happiness prediction, and model integration. The section explicitly discusses the limitations of previous studies and outlines how our approach addresses those gaps. We believe this adequately sets the stage for our study's contributions.
5. **Unclear Methodology Flow**
   *Comment:* Methodology in Section 2 does not follow a logical flow.
   *Response:* We have restructured Section 2 to follow a more logical flow. The subsections have been reorganized to ensure a step-by-step presentation of the methodology, making it easier for readers to follow the analysis process from clustering to model integration.
6. **Disconnected Statistical Analysis**
   *Comment:* The statistical analysis in Section 3 seems disconnected from the approach.
   *Response:* We believe that the statistical analysis is directly connected to the clustering and prediction steps. We have revised the section to explicitly reference the clustering results and how they inform the subsequent analysis, ensuring greater clarity.
7. **Intertwined Discussion & Results**
   Results (Section 3) now present findings objectively, while Discussion (Section 4) interprets implications, limitations, and policy recommendations without overlap.
8. **Unclear Degrees of Freedom**
   Table 4’s degrees of freedom (df=2) reflect the three-cluster ANOVA design (k-1=2). This is clarified in the caption and text.
9. **The Integrated Model Is Not Clear**
   *Comment:* The integration mechanism between the prediction and clustering models is not adequately explained.
   *Response:* We believe the integration mechanism is well explained in the manuscript. In the revised version, we have expanded the explanation of how clustering results influence the machine learning models by providing concrete examples and linking the clustering outcomes with model performance.
10. **General Lucidity**
    Jargon has been minimized, and technical terms (e.g., “elbow method”) are defined. The manuscript has been edited for logical flow and readability.

**Reviewer #2 Comments**

1. **Lack of Novelty**
   *Comment:* The study does not introduce novel methodologies or frameworks.
   *Response:* While the techniques used (K-Means clustering, Random Forest, and XGBoost) are established, the novelty lies in the integration of these methods in a hierarchical framework. This combined approach enhances predictive accuracy, a contribution we believe has not been adequately explored in existing literature. Furthermore, the incorporation of clustering results into machine learning models is an innovation in the study of global happiness.
2. **Lack of Explainability**
   *Comment:* The paper does not adequately discuss explainability techniques.
   *Response:* We agree that explainability is an important aspect, especially in machine learning models. In the revised manuscript, we have added a detailed discussion on feature importance, which explains how different factors like GDP and social support contribute to the happiness index prediction.
3. **Arbitrary Cluster Selection**
   *Comment:* The selection of three clusters seems arbitrary.
   *Response:* The number of clusters was determined using several established methods: the elbow method, silhouette method, and gap statistics. We have clarified this process in the manuscript and emphasized that the final choice of three clusters is backed by these statistical techniques, which support the robustness of our decision.
4. **Incomplete Variable Consideration**
   *Comment:* The study only considers economic and social factors, neglecting psychological, environmental, and cultural variables.
   *Response:* While we recognize the importance of psychological and environmental factors, the focus of this study was to explore the economic and social variables due to their strong theoretical and empirical backing in the literature on happiness. Expanding the scope to include psychological and environmental factors would be valuable in future work.
5. **Lack of Ablation Studies**
   *Comment:* The authors could have done ablation studies.
   *Response:* We appreciate this suggestion. However, due to time constraints, we were unable to conduct full ablation studies. Nonetheless, we have included an analysis of the importance of different variables in the model's performance, which provides insight into their relative impact on the prediction accuracy.
6. **Cross-Validation**
   Due to space constraints, we used other methods to verify the model
7. **Generic Policy Implications**
   *Comment:* The discussion on policy implications is too generic.
   *Response:* We have expanded the policy implications section to provide concrete recommendations tailored to specific clusters. The revised version now discusses actionable policies for high, medium, and low happiness countries, with recommendations for each group based on their unique characteristics.

We hope these revisions address the concerns raised by the reviewers and look forward to your feedback. Thank you again for your constructive comments, which have greatly contributed to improving this work.
