# Supplementary material for: Analyzing and predicting global happiness index via integrated multilayer clustering and machine learning models
Source: PLoS One. 2025 Apr 30;20(4):e0322287. doi: 10.1371/journal.pone.0322287 (PMC12043169; doi:10.1371/journal.pone.0322287)
Supplement: Attachment — Submitted filename: Response_to_Reviewers_auresp_2.docx [file pone.0322287.s004.docx]

**Response to Reviewers**

Dear Editors and Reviewers,

Thank you for your constructive feedback on our manuscript. We appreciate the opportunity to revise the paper and address the concerns raised. Below, we provide a point-by-point response to the reviewers’ comments and outline the revisions made to the manuscript.

**Reviewer Comment 1**:
*“The research framework should be smaller, and please watch out for arrows that don't align well in the left part of the figure. It would be nice if the figure had a sequence of steps.”*

**Response**:
We sincerely appreciate this observation. As requested,

1. **Figure 1 (Research Framework)**: We have revised the figure to ensure proper alignment of arrows in the left section and adjusted the layout to emphasize a clear sequence of research steps (e.g., “Data Processing → Clustering → Model Building → Validation”).

**Reviewer Comment 2**:
*“All the figures look blurry. Please insert them in the final version to see the full picture (pending acceptance).”*

**Response**:
All figures in the manuscript have been converted to high-resolution TIFF format to ensure optimal visual clarity.

**Reviewer Comment 3**:
*“The authors should spare separate sections for discussion, limitations or threats to validity, and impact of the study. The research objectives addressed in section 1.3 are expected to be discussed. The study's limitations, mainly found in many machine learning papers, should be described with their respective mitigation approaches.”*

**Response**:
We have restructured the manuscript to include dedicated sections addressing these concerns:

1. **Section 5: Discussion**
   - Added an in-depth discussion of the three research objectives outlined in Section 1.3, including:
     - Cluster analysis results (e.g., ANOVA-confirmed group differences in GDP, social support).
     - Comparative performance of machine learning models (Random Forest vs. XGBoost).
     - Policy recommendations aligned with UN Sustainable Development Goals.
   - Highlighted limitations of cluster analysis (e.g., data gaps in low-income countries) and proposed mitigation strategies (e.g., integrating satellite data).
   - Discussed nonlinear relationships between variables (e.g., social support’s stronger correlation with happiness than GDP).
2. **Section 6.3: Limitations and Mitigations**
   - Added four subsections addressing key limitations:
     - **Data Representativeness**: Acknowledged gaps in low-income regions (e.g., South Sudan) and proposed field surveys/satellite data integration.
     - **Temporal Dynamics**: Noted static model limitations and suggested longitudinal time-series analysis.
     - **Variable Selection Constraints**: Discussed omitted cultural/environmental factors and proposed text mining for cultural indicators.
     - **Clustering Assumptions**: Addressed K-Means’ spherical cluster bias and recommended hybrid clustering approaches.
   - Each limitation is paired with methodological or future research mitigations (e.g., interdisciplinary collaborations, advanced imputation techniques).

We believe these revisions comprehensively address the reviewers’ concerns and significantly strengthen the manuscript’s rigor, clarity, and impact. Thank you again for your valuable feedback. We are happy to provide further clarifications if needed.

Sincerely,
Dr. Yang
Corresponding Author
